# Ruminations on the Trigeminal Nerve
*Patrick Watson-Williams Memorial Lecture, University of Bristol, 18th Jan., 1964.


**Published:** 1964-07

**Authors:** Victor Lambert

**Affiliations:** Professor of Otolaryngology, University of Manchester


### RUMINATIONS ON THE TRIGEMINAL NERVE*

BY

VICTOR LAMBERT, M.D., CH.M., F.R.C.S.

*Professor of Otolaryngology, University of Manchester*

I count it a great honour to be keeping fresh the memory of one of the many distinguished otolaryngologists that Bristol has produced. I cannot recall that I ever saw Patrick Watson-Williams, though I know his son, and now his grandson is one of my colleagues on the staff of the Manchester Royal Infirmary. Patrick Watson-Williams belonged to the golden age of British Otolaryngology; an age on the pages of whose history occur such names as Sir St. Clair Thomson, J. S. Fraser, Jenkins, Scott, Brown-Kelly and Paterson. They are now numbered amongst the immortals of our specialty.

Whenever I think of Patrick Watson-Williams, I naturally think of the para-nasal air sinuses and perhaps the sphenodial air sinus more than any of the others. When one recalls that all the sensory nerves supplying the structures of the face pass close to the sphenoidal air sinus, it is not a very big step for one's interest to become centered on the trigeminal nerve.

### THOUGHTS ON EVOLUTION

One of the important stages in evolution was the appearance in the world of little furry animals. Initially their fur was a mechanism to keep these creatures warm; not just warm in the sense of producing the pleasant sensation which comes of warmth, but a means of conserving the body heat, because these little animals were at considerable risk through heat loss owing to the large surface area in relation to the body mass. The loss of body heat in these quickly moving animals, with their high metabolic rate, would have spelt disaster to the species.

The hairs throughout the body are related to the sense of touch, but some of them become more highly developed and more specialized, and appear as the structures which we know as vibrissae. In terrestrial animals and in the lower animals, these are found in specialized areas about the head, and in tree climbing animals on the ulnar surface of the fore-limbs. For our purpose we need only concern ourselves with the vibrissae occurring about the face and head of such animals. They receive their nerve supply from the trigeminal nerve, and in the one primitive mammal of which I have any knowledge the large size of the maxillary division of this nerve is most striking; this is in *Dasycercus cristicauda*, a practically pouchless Australian marsupial, whose curious tympanic structure the late Professor Wood-Jones and I described in the late 1930's. The large size of the nerve is a measure of its importance.

The hair follicles that give rise to vibrissae are well advanced in development even before the nerve supply to the skin has reached the skin surface. They reach a high grade of development long before the ordinary hairs of the skin. The vibrissae are present at a very early stage in embryonic life. There are many groups of vibrissae arranged about the head and face. The longest group are those of the upper jaw, and therefore represent the advance guard of the moving animal. The tactile impressions conveyed to the brain must therefore be fourth in order of importance, if vision, olfaction, and hearing are ranked as the first three.

We must also recall that the hairless, moist snout is supplied by the maxillary nerve as well. The hairless, moist snout is important in animals which hunt by smell, as it helps the animal to detect the direction of the wind. Man uses the same principle

*Patrick Watson-Williams Memorial Lecture, University of Bristol, 18th Jan., 1964.

when he wets his forefinger, the better to detect the direction and force of slight wind movements in the atmosphere.

When Man's ancestors took to the trees and the forelimb was no longer needed to support the body weight, it became the important explorer and tester of the physical environment. With such a change in status, the hand displaced the snout and vibrissae. This marked the decline in the importance of the trigeminal nerve as a defensive or survival mechanism. In respect of this, I. Maclaren Thompson has this to say: "Wood Jones points out that the important tactile function of the fifth nerve has been transferred to the median". Thompson further states, "that there is some confirmation of the belief held by certain anthropologists, that the jaw and snout region is one of those parts of the human body which is actually undergoing regressive evolution at the present time. The trigeminal nerve then has not only lost its importance as a great pathway of tactile sensation, but the actual territory innervated by it has been reduced. It is well known that structures which undergo evolutionary regression manifest an interesting susceptibility to pathological processes—may there not be some connection between the regression of the fifth nerve within the primate period of human phylogeny and the existence of trigeminal neuralgia."

### ANATOMICAL RELATIONS

The trigeminal nerve is, of course, a mixed nerve composed of motor and sensory fibres, but for the purpose of today's talk I want to concentrate on the sensory function only. The main sensory nucleus is situated in the pons. Its connections inside the brain are very extensive; more extensive than those of any other cranial nerve; and they range from the mesencephalon above to the level of the second cervical nerve below. The main nerve trunk appears on the surface of the pons nearer to its superior than its inferior border, in the same line as the facial, glosso-pharyngeal, and vagus trunks. Arising in the posterior fossa, the trigeminal nerve is recognized in the middle fossa by the conspicuous semilunar ganglion which lies in the trigeminal fossa, a shallow depression on the anterior surface of the petrous temporal, close to its apex. It is this close relationship between the Vth nerve and the petrous apex which is responsible for facial pain in suppuration of the petrous apex, which sometimes arises as a complication of suppurative disease of the middle ear.

From the ganglion the threefold branches appear, each going its separate way to become a thread in the composite clinical tapestry which we know as "facial pain". Every single nerve fibre in each of the three divisions finds its way, according to its heritage and destiny, to a tooth, a portion of the lining of the nasal sinus or some part of the skin surface of the face.

The cause of facial pain would be difficult enough to elucidate if the problem were limited to the basic anatomy of the trigeminal nerve in its three divisions; yet all its branches make contact with other cranial nerves and components of the sympathetic nervous system as well, and this adds to the riddle of it. The linking up and overlapping of the peripheral portion of the Vth nerve with the other cranial nerves, as well as the sympathetic has already been pointed out; one must also keep in mind the widespread connections which exist between the trigeminal nucleus and other structures within the brain. Only by doing so can we save ourselves from any pitfalls with which the diagnosis of facial pain is beset.

It is not for a rhinologist and certainly not for an occasion such as this to become involved in the problems which most correctly belong to the field of neurology and neurosurgery. The facial neuralgias of the migrainous type, tic doloreux and the facial pains of central origin, the rhinologist must know about in order to direct his patient into the correct channel for treatment. For too long and too often has my specialty been accused of operating too readily on the air sinuses in cases of facial pain. Our dental colleagues have suffered from the same kind of criticism.

### CLINICAL EXPERIENCES

Looking back on my professional life as an oto-rhinolaryngologist I have come to this conclusion, that there are four sites in our specialty which I call the graveyards of professional reputations. By this, I mean that early diagnosis of disease affecting these areas is difficult, unless the mind is kept constantly alert to sinister significance of seemingly minor symptoms. In this group I count the cerebellar-pontine angle, the hypopharynx, the subglottic region of the larynx and, finally, the post-nasal space or perhaps better known as the naso-pharynx. It has been in these situations that I have experienced the greatest number of wrong or late diagnosis, and let me be frank, I have had my share of them.

The post-nasal space is the area which lies behind the nasal passages; its roof is a part of the base of the skull; its posterior wall is formed by the anterior surface of the first two or three cervical vertebrae, anteriorly the posterior part of the nasal passage. Below and in front is the soft palate. It is roughly cuboidal in shape and about the size of a walnut. The post-nasal space can be the most trying region to examine and, even in the most skilled hands, the recognition of any disorders including small tumours can be most difficult. Neoplasms here consist, for the most part, of carcinomata and sarcomata with, very occasionally, those clinical rarities such as the juvenile naso-pharyngeal fibroma, the chordomas and the tumours arising in connection with Rathke's pouch.

Post-nasal tumours present in various ways and this is the order of frequency I established, in a survey I carried out some five or so years ago at the Christie Hospital and Holt Radium Institute, on 221 cases:—

1. Lymph gland enlargement.
2. Nasal symptoms, in order of their frequency:
   (a) nasal obstruction.
   (b) epistaxis.
   (c) nasal discharge.
3. Neurological:
   (a) neuralgic pain is easily the first in this group.
   (b) cranial nerve palsy—a poor second.
4. Otological:
   (a) deafness.
   (b) discharge.
5. Ophthalmological:
   (a) diplopia.
   (b) proptosis.
   (c) blindness.

A striking feature of this survey was the way in which patient and doctor tended to be much more complacent about neuralgic pain than about lumps in the neck caused by metastatic deposits from the primary naso-pharyngeal tumour.

Erik Godtfredson, in 1944, published a masterly survey of 454 cases of malignant naso-pharyngeal tumours and established the fact that 70 per cent of these had involved the Vth nerve, and a third of these patients first complained of neuralgic pains.

The practical point in all this is that a small tumour, which may never produce a recognizable bulging of the walls on the naso-pharynx can, by bursting through the pharyngeal fascia, gain the carotid sheath and spread upwards to the jugular foramen and involve the IXth, XIth and even the XIIth nerves, and also the cervical ganglion.

But more important to us than all this is the upwards spread along the carotid artery and through the foramen lacerum medium, thus approaching the posterior part and root of the Gasserian ganglion and through to the cavernous sinus.

In normal clinic practice the rhinologist uses the reflected light from the post-nasal mirror to examine this "round the corner" cavity we have alluded to. If this fails, he may use a nasal endoscope introduced *via* the nasal passages or the specially designed Yankauer's post-nasal speculum introduced into the cavity *via* the mouth and under the soft palate. This is usually carried out under a general anaesthetic.

If all these methods fail to help in deciding whether there is disease in the post nasal space and, if there is, its exact nature, the surgical approach to the cavity as described by C. P. Wilson must be carried out. This consists of freeing the soft palate from the hard palate and displacing it, so giving a wide approach to the post-nasal space, not only for easy and accurate examination, but also to enable samples of tissue to be removed for microscopic examination. The operation produces little discomfort and no permanent upset of palatal function. A heavy responsibility rests on the shoulders of the rhinologist, when he is the sole judge of whether or not malignant changes are present in the post-nasal space. The use of Wilson's approach will help him to offer an opinion with greater confidence and certainty.

In 1943 a female of 18 years came into the out-patient clinic complaining of pain in and around the right eye and forehead. The pain was paroxysmal and associated with spectra in the eye as well. The condition had been present for three years and sub-mucous resection had been performed some months previously, with no relief from her symptoms. On going more closely into her story, she admitted to pain in the right ear and an increasing amount of pain over the three divisions of the trigeminal nerve. The patient produced an X-ray of the air sinuses, which had been taken in another centre, but no sinus disease could be recognized. There was, however, an obvious and grossly impacted lower wisdom tooth on the side of her trouble. This did not seem to us to be the cause of her troubles. On close inspection there did appear to be something unusual in the bone of the floor of the middle fossa. This finding seemed to be more in keeping with a tentative diagnosis of post-nasal malignancy, as suggested by my colleagues who had referred the patient to me.

Dr. Blair Hartley of the X-ray diagnostic side of the Christie Hospital suggested that a basal projection radiogram should be taken. This gave us the answer, because the foramen ovale appeared to be grossly enlarged to a circle of 4 cm in diameter and the foramen rotundum was enlarged as compared with one on the opposite side. I sent the patient to Sir Geoffrey Jefferson's clinic. He commented on the clear cut nature of the enlargement, so completely different from the ragged enlargement produced by invasion due to a post-nasal malignant tumour. The nature of the history, plus the sensory changes he found over the trigeminal area and the clear-cut skull erosion, left him in no doubt that here was neurinoma of the trigeminal nerve. At operation this was confirmed, and the tumour was successfully removed. The patient was known to be fit and well 10 years after operation. This case is one of the seven reported by Sir Geoffrey Jefferson at the Congress of Neurosurgeons in New Orleans in 1953. This patient's story illustrates not only the difficulty in the diagnosis of some cases of facial pain, but the interest the condition can be to many branches of our profession.

Some two years after this case had come my way, another patient was sent to me by a consultant physician, with much the same story but without any changes in sensation over the trigeminal area. She had already had several antral lavages without the pain being cured, though there is no doubt that antral sepsis had been present. Fresh X-rays were taken including a basal projection view owing to its help in the other similar case. Again, on the right side was a clear-cut erosion in the base of the skull, but some distance in front of the foramen ovale. At that time, I was interesting myself in the anomalies of the sphenoidal air sinus and I wondered if this could be one, although I still had the memory of the neurinoma in my mind. I tried to introduce radio-opaque medium into the sphenoidal sinus by the Proetz displacement technique during an actual neuralgic crisis, without success. During a pain-free interval some days later, the lipoidal was not only accepted by the sphenoid sinus, but outlined the suspected area, which proved to be a pterygoid extension of the sphenoidal sinus. At a subsequent date I explored the air sinus, more in hope than in expectation of curing the patient. The lining of the sinus, which I removed and had sectioned, did show some inflammatory thickening. The aggressive rhinologist would, I am sure, call this real pathology. I have always had my doubts. There was some symptomatic improvement for a time but not a cure.

The rejection of the lipoidol in the acute painful stage has both puzzled and interested me, the more since reading McAuliffe and his co-workers' observations on "Pain in Diseases of the Nasal Sinus." They regard the sinus linings as less pain-sensitive than the mucosa covering the approaches, such as the sinus ostia and the turbinals, and say that trigeminal pain which is not controlled by the reduction of turbinal congestion by local analgesia, is not due to nasal or paranasal inflammation.

There comes a time to all of us who are in any way interested in trigeminal or facial pain, when we have to give serious consideration as to whether we ought to carry out some form of trigeminal interruption to control the symptoms. This, I feel, is a problem for the neurosurgeon. He must decide when and by what means this must be done. In the painful terminal stage of post-nasal malignancy, it can be very much a worthwhile procedure to try, in the first place, alcohol injection of the ganglion. Should this fail, a root section through the posterior fossa approach should be carried out and the IXth nerve can be divided at the same time.

There is and perhaps always will be some facial pain for which we cannot find a cause. Is this any wonder when we think of the vulnerability of the trigeminal nerve because of its long course, leaving out of consideration its central and peripheral minglings with other nerves?

The importance of the central connection of the sensory nucleus of the trigeminal nerve is illustrated in a case which the late Lord Stopford brought to my notice. It may be recalled that Lord Stopford was our Professor of Anatomy in the University of Manchester and a fine neurologist, before he became the Vice-Chancellor of the University and subsequently to be rewarded for his outstanding work by a life peerage. The patient, a man in middle life, was suffering from severe facial pain, which was constant in character, and over the distribution of the trigeminal nerve. Full investigation revealed an encysted abscess in the lower part of the thoracic cavity, fairly low down. When this was evacuated the pain disappeared. The cause of this unusual referred pain was thought to be that the patient had a low spinal Vth sensory nucleus and that his phrenic nerve had a high origin, and that the pain was due to stimulation of afferent fibres in the phrenic, spilling over to the lower sensory nucleus of the trigeminal nerve. I also recall a patient of mine whose initial symptoms of a coronary occlusion was a very violent attack of trigeminal neuralgia which lasted some 24 hours before a cardiac lesion was detected.

In conclusion I would like to quote Professor Norman Dott as saying that in his experience, and this is considerable, he has never seen one case of truly psychogenic facial pain. This is a sobering thought. I recall that one of my teachers would constantly remind us that even a neurotic had to die of something. It is a wise admonition not to shelter our ignorance and limitations behind that over-used word "functional". Until we have a means of measuring pain with anything like the ease of measuring body temperature we must depend to a large extent on the veracity and powers of description of our patients, and on most occasions they will be telling the truth.

